# A Compendium for Novel Marker-Based Breeding Strategies in Eggplant

**DOI:** 10.3390/plants12051016

**Published:** 2023-02-23

**Authors:** Luciana Gaccione, Matteo Martina, Lorenzo Barchi, Ezio Portis

**Affiliations:** Department of Agriculture, Forest and Food Sciences (DISAFA), University of Turin, 10095 Grugliasco, Italy

**Keywords:** eggplant, *S. melongena*, QTLs, candidate genes

## Abstract

The worldwide production of eggplant is estimated at about 58 Mt, with China, India and Egypt being the major producing countries. Breeding efforts in the species have mainly focused on increasing productivity, abiotic and biotic tolerance/resistance, shelf-life, the content of health-promoting metabolites in the fruit rather than decreasing the content of anti-nutritional compounds in the fruit. From the literature, we collected information on mapping quantitative trait loci (QTLs) affecting eggplant’s traits following a biparental or multi-parent approach as well as genome-wide association (GWA) studies. The positions of QTLs were lifted according to the eggplant reference line (v4.1) and more than 700 QTLs were identified, here organized into 180 quantitative genomic regions (QGRs). Our findings thus provide a tool to: (i) determine the best donor genotypes for specific traits; (ii) narrow down QTL regions affecting a trait by combining information from different populations; (iii) pinpoint potential candidate genes.

## 1. Introduction

Eggplant (*Solanum melongena* L., 2n = 2x = 24) is the fourth most important crop economically and nutritionally, belonging to Solanaceae, a large plant family including important crops such as tomato, potato, pepper, and tobacco. According to the latest FAOSTAT report [1], eggplant is cultivated worldwide, with a global production of 58 Mt in 2021. China and India are the main producing countries, accounting for about 86% of total production, while Egypt, Turkey, and Italy represent the main producers of the Mediterranean region. Contrary to most other solanaceous crops originating in the New World [2,3,4,5,6,7], eggplant has a phylogenetic uniqueness due to its exclusive Asian origin. The species has been reported to be the result of two or three independent domestication events, though recent studies have suggested a unique one [8,9]. Within the genus, eggplant and its relatives belong to the subgenus *Leptostemonum*, collectively known as the ‘spiny solanum’ group [10]. The most closely related species from the eggplant clade have been reported to be the direct wild ancestor *S. insanum* L. and the sister species *S. incanum* L. [11,12], while two other eggplant crops belonging to the Anguivi clade, the Ethiopian/scarlet eggplant (*S. aethiopicum* L.) and the African/Gboma eggplant (*S. macrocarpon* L.), have a locally important production, with the fruits and leaves of both species used for food and medicine [10,13,14]. Compared with cultivated eggplants, their wild relatives present a broader adaptation to the environment and climate, carry abundant genetic diversity, and have higher potential in crop improvement [15,16].

A necessary condition to exploit the introgression of traits of interest from crop wild relatives (CWRs) into cultivated plants is the knowledge of the associated or responsible genes/quantitative trait loci (QTLs) controlling the traits [17,18,19,20,21]. To dissect the genetic basis of complex traits, genomic studies using bi-parental QTL mapping (linkage mapping) and genome-wide association (GWA) mapping can be conducted, based on the significant association between markers and a phenotype of interest. Traditional biparental mapping approach is highly dependent on the genetic diversity of the two parental lines, and the effects of the detected QTLs vary depending on the chosen population [22,23]. Therefore, the number of genetic recombination events that occur during the construction of the mapping population affects both genetic mapping resolution and allele richness. The construction of a genetic linkage map requires a mapping population to analyze the recombination of specific molecular markers defining the position and relative genetic distance of the markers along the chromosomes. In the past few decades, several first-generation genetics’ maps (based on pre-NGS techniques) were developed from interspecific hybridizations between cultivated *S. melongena* and *S. linnaeanum* or *S. incanum* and applied for QTL analyses of domestication and morphological traits [24,25,26], as well as to locate genes involved in polyphenol biosynthesis [27]. Intra-specific maps were also constructed using both F2 and DH populations [28,29,30,31,32,33]. In parallel with the advances in the genetic linkage maps, the identification of QTL regions associated with agronomic traits has been considerably promoted in eggplant. The first NGS-based eggplant genetic map was developed on an intra-specific F2 population using RAD-tag derived markers [34] and genotyped via Illumina GoldenGate^©^ assay [35,36]. Afterward, several genetic linkage maps were constructed for mapping disease resistance, parthenocarpy, and plant morphological-related traits [37,38,39,40,41,42,43,44,45,46,47,48,49,50,51,52]. Recently, a multiparent advanced generation intercross (MAGIC) population was developed by Mangino et al. [53], allowing the identification of putative regions and candidate genes for anthocyanin pigmentation.

In contrast, GWA studies are performed on a population of unrelated individuals in a heterogeneous collection, in which historical recombinations have accumulated over generations. As a result, the association mapping shows a higher map resolution and greater number of investigated alleles compared with the QTL mapping approach [54]. The detection power of the GWA approach can be affected by many factors including the population structure and dimension, allele frequency, as well as the phenotypic variation [55]. Furthermore, population structure (i.e., genetic relatedness between individuals in a population) may lead to false-positive associations between genotypes and the investigated traits if not taken into account [56,57,58]. For these reasons, an integrated approach may be crucial for the understanding of the architecture of complex quantitative traits. Nowadays, the availability of large germplasm collections, together with relatively low genotyping prices, provide robustness to GWA studies, making it possible to understand the architecture of complex traits [59]. However, only a few association mapping studies have been reported in eggplant. A first attempt was conducted by Ge et al. [60] to identify functional genes and QTLs related to fruit-related traits using a panel of 141 eggplant accessions. Afterwards, a larger panel (191 accessions) was employed to analyze the marker/trait associations for key breeding fruit and plant traits [61,62,63]. The described association mapping studies not only highlighted numerous previously identified genes/QTLs, but also allowed the discovery of novel loci and candidate genes, providing a valuable resource for the development of a marker-assisted selection breeding strategy.

A next step in the genomic era is provided by the concept of pangenome, the nonredundant set of genomic sequences within a species which include the core genes present in all individuals and dispensable genes only found in a subset [64]. Furthermore, pangenome approaches allow the identification of selective sweeps, presence/absence variations (PAVs), and structural variations linked to key agronomic traits. Compared to tomato and pepper, for which pangenome studies have been carried out using hundreds of accessions [65,66,67], the first eggplant pangenome has been recently established from 24 accessions of *S. melongena*, one accession of *S. insanum*, and one accession of *S. incanum* [68].

All the above-mentioned approaches have allowed the identification of a wide number of eggplant QTL regions for many agronomic and quality-related traits. To provide a comprehensive overview of the current genetic knowledge, 28 scientific papers and their supplemental data were collected here, integrated, and summarized. Combined information represents a valuable tool for marker-assisted selection breeding schemes, since it may be employed to find potential donors for a particular trait, to highlight key QTL regions as well as potential candidate genes for clarifying the genetic architecture of the eggplant agronomic traits.

## 2. Construction of a Unified Eggplant QTL Map and Identification of Candidate Genes

A total of 28 scientific articles reporting both QTL mapping and GWA studies were analyzed (Table S1). The studies were selected based on the feasibility of retrieving the markers’ position on the genome. This is an issue for the research carried out before a reference genome came out, where just the genetic linkage positions (cM) of markers were reported, or dominant markers as AFLP were used. To overcome this constraint, the physical position of the markers (when possible) was retrieved by aligning their sequences on the eggplant reference genome (line ‘67/3′-version 4.1) [68] using BLASTn [69]. If markers were instead mapped to another genome [14,38,70,71], minimap2 [72] was employed to determine their position on the eggplant reference genome v4.1. All the collected QTLs data were organized in a single database (Table S2) including information on QTL name, QTLs, related marker ID, chromosome position (in cM and Mb), significance of the associations with the examined trait (*p*-value; LOD score; percentage of variation explained by the QTL and effect), and the mapping population (type, cross, or association panel). Eggplant traits included in this review were classified into seven classes: (i) morphological traits—including plant (PL), leaf (LF), and flower (FL) traits; (ii) prickles (PK); (iii) parthenocarpy (PT) and male sterility (MS); (iv) fruit-related traits—including shape (SH), productivity (PR), quality (QL), and metabolites (MT); (v) anthocyanins (AN); (vi) biotic resistance (pathogens and pests’ resistance) (RS); (vii) abiotic resistance. Furthermore, to suggest genomic regions harboring potential candidate genes, quantitative trait regions (QGRs) were retrieved from overlapping QTLs for each eggplant chromosome, and associated candidate genes were identified in literature. To overcome the absence of a reported genetic confidence intervals or (average) *linkage disequilibrium* (LD) decay for the collected QTLs, potential positional errors were standardized by setting an empirical defined window of ±2.5 Mb around the QTLs, as suggested by Martina et al. [73]. QGRs were named based on the trait classes defined above. Because no QTLs were available for male sterility and pests/abiotic resistances, the literature was investigated to identify potential candidate genes (Table S3).

## 3. Morphological Traits

**Figure 1:**
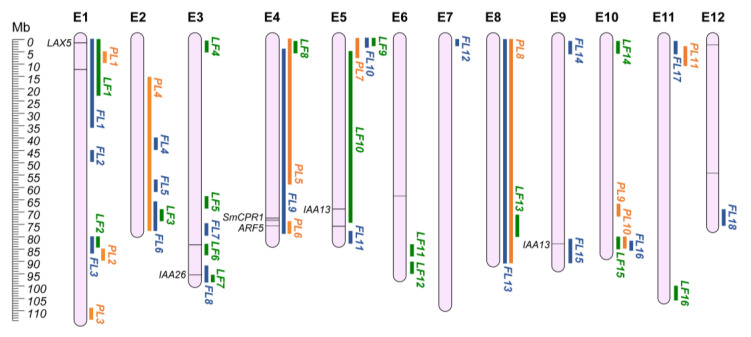
Morphological QGRs chromosome map (E1–E12). Orange: Plant QGRs (PL); Green: Leaf QGRs (LF); Blue: Flower QGRs (FL). Main candidate genes are reported in italics, while information about the other candidates (represented by bars on the chromosomes) are shown in Table S3.

Eggplant is a bushy and vigorous plant, with large leaves and woody stems, and several morphological traits can be targeted during varietal development to obtain cultivars with higher agronomical characteristics. For instance, height impacts on plant habit and on the agronomic management of the crop, while leaf shapes, length, and width are crucial for photosynthetic efficiency. Flower shape, together with its sexual organs’ development, is directly linked to flower fertility, and thus to plant productivity. Several QTLs have been reported for the main morphological traits in eggplant. To ease the search for regions of interest, QTLs were split into three categories: (i) plant (PL); (ii) leaf (LF); and (iii) flower (FL). Data collection revealed a total of 84 morphology-related QTLs. By comparing these regions, 45 unique QGRs were defined (Table S2; Figure 1). On the whole, 38 QTLs were not included in any QGRs due to the lack of their genomic position.

### 3.1. Plant (PL)

Plant-related traits can influence agronomic strategies for crop production. For example, by decreasing the plant height, the growing habitus of the plant can be changed, allowing different cultural methods and potentially improving the field productivity. No clear candidate genes have been reported in the literature for these traits, except for *SmCPR1* (SMEL4.1_04g017430.1), a cytochrome P450 reductase putatively associated with plant dwarfism [74,75] and located on QGR PL6 (chr. E4).

### 3.2. Leaf (LF)

Understanding the mechanism of leaf development is essential to improve crop management, influencing plant productivity and stress tolerance. Small leaf mutants (slf) have been recently generated by ethyl methane sulfonate (EMS) mutagenesis [76]. Transcriptomic analysis indicated a dominance of the auxin signal during leaf development in mutated plants, allowing the identification of *AUX1* (annotated as *LAX5* in v4.1-SMEL4.1_01g003480.1), *ARF5* (SMEL4.1_04g022210.1), and three Aux/IAA (SMEL4.1_05g020420.1, SMEL4.1_09g022160.1, and SMEL4.1_03g032430.1-QGR LF7) genes as potential candidates for the observed phenotype. The latter were proposed to be the main genes responsible for leaf growth and morphogenesis in the obtained mutants.

### 3.3. Flower (FL)

Sexual organs’ characteristics, such as ovary length, diameter, and hairiness, impact on the possibility of the flower being pollinated. Two QTLs hotspots were identified in QGR FL1 (chr. E1) and FL6 (chr. E2), associated with ovary length, ovary diameter, ovary hairs, flower shape, and peduncle length. A comparative proteomic analysis allowed the identification of differentially expressed proteins in heterostylous pistil development [77], highlighting the potential role of nine genes (Table S3) during flower development. Additionally, some proteins associated with programmed cell death were associated with S-morph pistils, belonging to flowers generally possessing a small and highly reduced gynoecium and lower productivity.

## 4. Prickles (PK)

**Figure 2:**
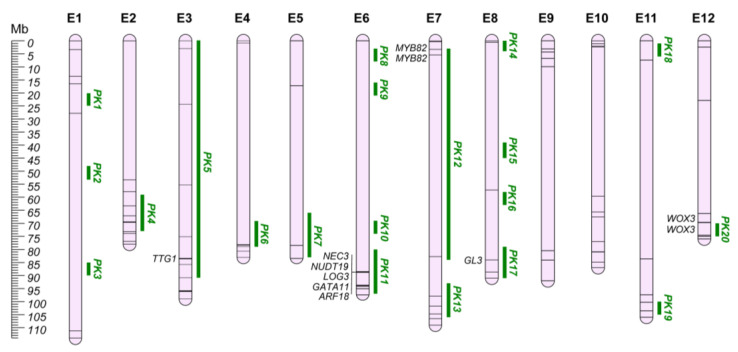
Prickles’ QGRs (PK; Green) chromosome map (E1–E12). Main candidate genes are reported in italics, while information about the other candidates (represented by bars on the chromosomes) are shown in Table S3.

Eggplant is the only solanaceous crop possessing a prickly phenotype. Prickles can be found on eggplant leaves, stems, and fruit calyxes and are modified glandular trichomes and cortical cells used as a defensive strategy against herbivore attacks, generally perceived as an undesirable commercial trait [78]. Many eggplant cultivars present prickles on the fruit calyx, since in certain world regions they are perceived of superior organoleptic quality, while the prickles on the vegetative tissues are generally absent, as the result of the positive selection in breeding programs [79]. Despite the several mapping studies that reported QTLs for this trait [24,27,42,62,63,80], the genetic basis of prickle formation in eggplant remains unclear. Data collection revealed a total of 115 QTLs and, on comparing these regions, 20 unique QGRs were defined (Table S2; Figure 2). The remaining nine QTLs were not included in the QGRs due to the lack of genomic position.

The QGR PK11, located on chr. E6, has been considered the main region responsible for eggplant prickles, and several mapping studies have located prickly related QTL in this region [24,42,79]. In addition, Portis et al. [63] confirmed such associations through GWA analysis. By fine-mapping the region (named PI locus), Miyatake et al. [80] delineated candidate genes encoding for: (i) carbonic anhydrases (SMEL4.1_06g026670.1, SMEL4.1_06g026680.1); (ii) nudix family hydrolases (SMEL4.1_06g026690.1); (iii) GATA transcription factor (SMEL4.1_06g026740.1); and (iv) Auxin Response Factor (SMEL4.1_06g026750.1). Furthermore, the PI locus-ascribed 0.5-kb deletion in the ‘Togenashi-senryo-nigo’ genotype was proposed as affecting the gene expression level of neighboring genes, particularly the downstream *GATA11* (SMEL4_06g026740.1). Furthermore, comparing prickled and non-prickled genotypes, a selective sweep (SS) harboring genes encoding *NUDT19* (SMEL4_06g026690.1), *GATA11* (SMEL4_06g026740.1), and *ARF18* (SMEL4_06g026750.1) was identified in the eggplant pangenome by Barchi et al. [68]. The SS on chr. E6 is close to the previously reported QTLs and QTNs, as well as the morphological marker *PRICKLINESS* [27] and the PI locus, suggesting a possible target for eggplant breeding improvement. In addition, the transcriptome analysis performed by Zhang et al. [78] identified the *ARF18* gene (SMEL4.1_06g026750.1), which was located in the PI locus, as the key responsible for the formation of prickles, providing new insights into the regulatory molecular processes driving prickles’ morphogenesis in eggplant.

Another major QTL affecting the prickles’ development in the plant was recently identified on chr. E12 (QGR PK20) [79]. This genomic region has been thoroughly investigated underlying seven putative candidate genes involved in the prickle’s formation. Among these, SMEL4.1_12g013270.1 and SMEL4.1_12g013280.1, encoding a WUSCHEL-related homeobox 3B protein (*WOX3*), were proposed as candidates influencing calyx prickle formation. Indeed, higher expression levels of SMEL4.1_12g013280.1 in prickly individuals, and a 22-bp deletion affecting the second exon of the same gene in prickleless individuals suggest that *WOX3* genes are likely involved in the development of calyx prickles in eggplant.

On chr. E7, two genomic loci controlling prickles’ traits have been identified (QGR PK12 and PK13). In particular, QGR PK12 was associated with an SS comprising two adjacent *MYB82* (SMEL4.1_07g003480.1 and SMEL4.1_07g003490.1), which are homologous of the Arabidopsis *GLABRA1* gene [68]. *GLABRA1* plays a pivotal role in the formation of a trimeric complex with GLABRA3/ENHANCER of GL3 (*GL3/EGL3*) and TRANSPARENT TESTA GLABRA1 (*TTG1*), which is essential for the positive regulation of *A. thaliana* trichome initiation [78,81,82,83,84]. Both the BHLH family protein *GL3* (SMEL4.1_08g024700.1) and the WD repeat family protein *TTG1* (SMEL4.1_03g019420.1) are also located in QGRs PK17 (chr. E8) and PK5 (chr. E3), respectively [78].

## 5. Parthenocarpy and Male-Sterility (PT and MS)

Crop reproduction is tightly connected to plant productivity and fruit quality. If sexual behaviors, such as male sterility (MS) and self-incompatibility (SI), can be employed for hybrids’ production, seedlessness, as a result of parthenocarpy, is particularly appreciated by consumers [85,86].

Male sterility (MS) consists in the failure of plants to produce functional anthers, pollen, or male gametes. Male sterile mutants are classified into (i) structural, (ii) sporogenous, and (iii) functional types, based on anthers’ development and phenotype [87]. Male sterility is a useful trait in breeding programs, facilitating the production of hybrid seeds and avoiding emasculation, and genic male sterility (GMS) and cytoplasmic male sterility (CMS) have been proposed as causative mechanisms of this trait [88]. A number of genes responsible for both CMS and GMS are known for many plant species [88,89,90,91,92], and in eggplant, several mutants manifesting GMS caused by recessive nuclear genes were reported [93], as well as genes involved in CMS [94,95,96]. In the last decade, different biotechnological strategies have been tested [97,98], differentially expressed genes (DEGs) were identified (Table S3) [99,100,101,102], and the protein–protein interactions of *SmCOI1* (SMEL4.1_05g001020.1) with *SmOPR3* (SMEL4.1_07g003350.1) and *SmJAZ1* (annotated as *TIFY10A* in v4.1-SMEL4.1_12g001970.1) were investigated [103,104], providing valuable information for the dissection of the genetic basis of male sterility.

**Figure 3:**
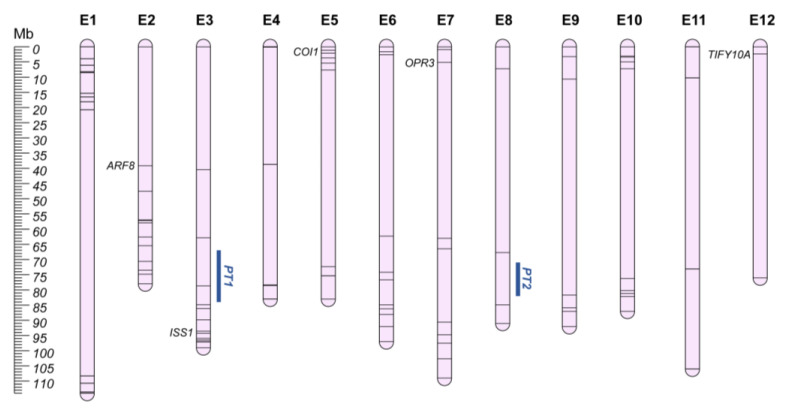
Parthenocarpic QGRs (PT; Orange) and Male Sterility chromosome map (E1–E12). Main candidate genes are reported in italics, while information about the other candidates (represented by bars on the chromosomes) are shown in Table S3.

Parthenocarpy (PT) is defined as the growth of the ovary into a fruit without pollination and/or fertilization, and results in the acquisition of seedless commercial varieties with a high fruit yield [105]. In eggplant berries, the presence of seeds causes a more intense and faster fruit pulp browning, due to oxidation of chlorogenic acid by polyphenol oxidases, and the biosynthesis of bitterness-related and flesh hardness-related compounds such as saponin and solasonin [106,107]. Furthermore, as sub-optimal environmental conditions negatively influence fruit yield and impact on reproductive processes (i.e., pollen formation, dispersal, germination, and fruit fertilization), parthenocarpic varieties represent a cost-effective solution to improve fruit set and growth in different environments [108]. In 2012, Miyatake et al. [109] investigated the genetic basis of parthenocarpy, reporting the trait as polygenic and identifying a major QTL on chr. E8 (~30% explained variability; Cop8.1; QGR PT2; Figure 3), and one on chr. E3 (Cop3.1; QGR PT1). Furthermore, several DEGs were reported (Table S3) [110], and *SmARF8* (SMEL4.1_02g004290.1) [111] and *Pad-1* (SMEL4.1_03g031670.1, annotated as *ISS1*) [112], an aminotransferase involved in auxin homeostasis, were recently highlighted as inducing parthenocarpy. The latter was reported to be mainly responsible for the *Pad-1* locus identified on chr. E3, 10Mb upstream QGR PT1.

## 6. Fruit-Related Traits

**Figure 4:**
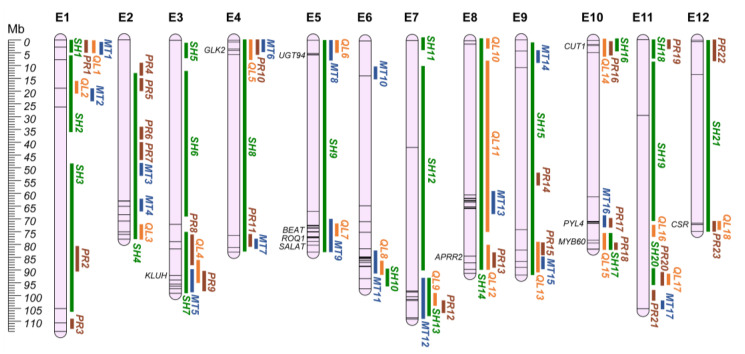
Fruit-related QGRs chromosome map (E1–E12). Green: Shape QGRs (SH); Brown: Productivity (PR); Orange: Quality (QL); Blue: Metabolites (MT). Main candidate genes are reported in italics, while information about the other candidates (represented by bars on the chromosomes) are shown in Table S3.

Plant productivity and fruit quality have been the main focus of plant breeding for decades. Indeed, production directly impacts on producers’ acceptance of novel lines, with traits such as fruit weight and number of fruits affecting total yield [113]. On the other hand, fruit quality perception by the consumer is no more merely related to a morphological focus (fruit shape, glossiness, presence of seeds in the berry, pericarp firmness, and chlorophyll pigmentation) but also to a nutritional one [114]. Most of the nutrient properties of the eggplant fruit are related to vitamins, phenolic compounds, especially chlorogenic and hydroxycinnamic acids and their conjugates, and other phenylpropanoids [115]. Anti-nutritional compounds, such as steroidal glycoalkaloids and polyamine conjugates, are accumulated both in the flesh and peel as a toxic defense mechanism against herbivores, providing a bitter taste to the fruits [116]. As several traits are involved in fruit quality, to ease the search for regions of interest, QTLs were split into four categories: (i) shape; (ii) productivity; (iii) quality; and (iv) metabolites. The comprehensive list of the traits included in each category can be found in Table S2. A total of 304 QTLs were used for the identification of 79 distinct QGRs (Table S2; Figure 4), while 41 QTLs had insufficient information to retrieve their physical position.

### 6.1. Shape (SH)

Twenty-one QGRs, identified by 162 QTLs, were associated with shape-related traits. In these regions, 40 candidate genes for fruit shape were identified (Table S3) [53,68,117,118], including the SUN, OVATE and YABBY gene families. In tomato and pepper, SUN and OVATE have been associated with fruit elongation [119,120,121], while a YABBY transcription factor has been reported to be involved in fruit size determination associated with the fas locus in tomato [122]. The SUN-associated protein is a positive regulator of growth and has been proposed to be involved in fruit elongation and hormones or secondary metabolite levels [123], while Ovate family proteins (OFPs) have been identified as encoders of a negative regulator of fruit growth [119].

### 6.2. Productivity (PR)

Fifty-four QTLs were identified, defining 23 QGRs related to productivity traits. The genetic basis of fruit weight is poorly understood in the Solanaceae family, and in tomato few genomic regions have been strongly associated with the trait, with almost no candidate genes identified [124]. Mu et al. [125] identified a mutant allele in cell-size regulator (*CSR*-Solyc11g071940-fw11.3) genes associated with the domestication of tomato fruit, assessing its expansion in the Solanaceae family. In eggplant, we identified SMEL4.1_12g014140.1 through ortholog-driven gene mining, associated with QGR PR23, on chr. E12 (annotated as At5g22090 in V4.1). Recently, Li et al. [126] identified *SlKLUH* (Solyc03g114940), a CUP78A that positively regulates fruit weight by increasing the number of cell layers in the pericarp [127], as being mainly responsible for QTL fw.3.2 in tomato. Furthermore, pangenome analysis [128] revealed a positive association between *SlKLUH* copy number and fruit weight in tomato. *CYP78A5* (SMEL4.1_03g027710.1) is its orthologous gene and is located in QGR PR9 (chr. E3). These two genes are, to our knowledge, the first candidates reported for fruit weight in eggplant.

### 6.3. Quality (QL)

Eighteen QGRs, identified by 42 QTLs, were associated with fruit quality-related traits. Six QGRs contained nine QTLs associated with chlorophyll pigmentation of the berry, generally corresponding with the presence of a flesh green ring after fruit cut and chlorophyll accumulation in the peel. Among the reported genes (Table S3), two were identified as the main genes responsible for chlorophyll-controlling regions on chrs. E4 and E8 [129]. Indeed, *GLK2* (SMEL4.1_04g003340.1-chr. E4-QGR QL5) and *APRR2* (SMEL4.1_08g020990.1-chr. E8-QGR QL12) have been reported to be promoters of chloroplast development in several solanaceous and cucurbitaceous crops regulating pigment accumulation [130,131,132,133,134]. For glossiness (i.e., accumulation of waxes in the berry epidermis) nine QTLs were identified in five QGRs (QL4, QL8, QL11, QL13, and QL15). In QGR QL15 (chr. E10), a *MYB60* (SMEL4.1_10g024240.1-formerly annotated as *MYB30*), a regulator of cutin and wax biosynthesis and cuticle development [135,136,137,138,139], was reported to be involved in the expression of a 3-ketoacyl-CoA synthase 6 (*CUT1*, formerly reported as *KCS6*; SMEL4.1_10g001780.1) [140], falling within the QGR QL14. KCSs are a family of synthases involved in the biosynthesis of very long chain fatty acids, playing a key role in wax biosynthesis [141]. KCSs mutants have been reported as producing lower amounts of cuticular wax in *Medicago truncatula* [142], *Arabidopsis* spp. [143,144,145], *Populus trichocarpa* [146], and *Brassica rapa* [147].

### 6.4. Metabolites (MT)

Seventeen QGRs, identified by 46 QTLs, were associated with metabolites’ accumulation in the berry. In these regions, six candidate genes were reported as potentially involved in the metabolism of steroidal glycoalkaloids (SGAs) and polyamine conjugates. On chr. E10, the abscisic acid receptor *PYL4* (QGR MT16; SMEL4.1_10g018120.1) was associated with pseudoprodioscin biosynthesis, while a UDP-glycosyltransferases (QGR MT8; *UGT94*; SMEL4.1_05g004840.1) was identified in a QTL for demissine on chr. E5. Four more genes were identified on chr. E5, potentially involved in polyamine conjugates accumulation: (i) TMV resistance protein N (QGR MT9; *ROQ1*; SMEL4.1_05g020480.1); (ii) acetyl-CoA-benzyl alcohol acetyltransferase (QGR MT9; *BEAT*; SMEL4.1_05g018170.1); (iii) an acyl-lipid (9-3)-desaturase (QGR *MT13*; SMEL4.1_08g012960.1); and (iv) a salutaridinol 7-O-acetyltransferase (QGR MT9; *SALAT*; SMEL4.1_05g020260.1), involved in alkaloid biosynthesis in *Papaver somniferum* [46].

## 7. Anthocyanins (AN)

**Figure 5:**
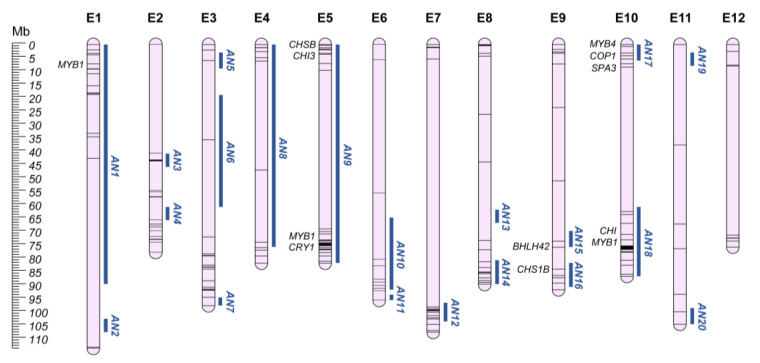
Anthocyanins QGRs (AN; Blue) chromosome map (E1–E12). Main candidate genes are reported in italics, while information about the other candidates (represented by bars on the chromosomes) are shown in Table S3.

Anthocyanins are an important class of flavonoids, glycosylated polyphenolic compounds that represent a vast class of plant pigments, with a range of color from orange to blue [148]. These plant secondary metabolites with high antioxidant capabilities play an important role in plant reproduction by attracting pollinators, protecting plants from several biotic and abiotic stresses. Anthocyanins’ accumulation avoids lipid peroxidation and maintains membrane integrity, lowering cell senescence, and improving vegetables’ postharvest performance [149]. In plants, the most common anthocyanins are derived from the metabolism of six anthocyanidins, namely pelargonidin, cyanidin, delphinidin, peonidin, petunidin, and malvidin [150]. In violet/black eggplant, as well as in pepper, the only anthocyanins reported to be accumulated are derived from delphinidin, which can also be present in the vegetative organs of the plants. In the fruits, the delphinidin level is higher at the unripe stage and decreases upon ripening to complete disappearance [115]. Delphinidin-3-(p-coumaroyl-rutinoside)-5-glucoside, commonly known as nasunin, is the most frequent anthocyanin structure in pepper and eggplant fruits [151]. In addition, some eggplant accessions have been observed accumulating a non-acylated anthocyanin, delphinidin-3-rutinoside [44]. The genetic control of anthocyanin biosynthesis, its distribution, and accumulation in Solanaceae species, including eggplant, has been extensively studied [152,153,154,155,156,157,158,159], and candidate genes included in the defined QGRs were retrieved from the literature (Table S3). The primary level of regulation for anthocyanin biosynthesis is the expression of regulatory and structural biosynthetic genes. Structural genes are classified as early (EBG; chalcone–flavonone synthase-CHS; chalcone–flavonone isomerase-CHI; flavanone 3-hydroxylase-F3H) and late (LBG; flavonoid 3′-hydroxylase-F3′H; flavonoid 3′,5′-hydroxylase-F3′5′H; dihydroflavonol 4-reductase-DFR; anthocyanidin synthase-ANS; flavonoid 3-O-glucosyltransferase-UFGT; flavonol synthase-FLS) biosynthetic genes [160,161,162]. Data collection indicated 153 QTLs as associated with anthocyanin levels in various organs, grouped in 20 QGRs (Table S2; Figure 5). Four papers additionally identified 17 QTLs that lacked information on their chromosomal position.

By focusing on highly QTL-dense QGRs (AN9, AN16, AN18), four structural genes, two CHS (SMEL4.1_05g000250.1 and SMEL4.1_09g023150.1) and two CHI (SMEL4.1_05g001480.1 and SMEL4.1_10g016630.1), were identified by several studies as closely associated with anthocyanin accumulation under different conditions. Moreover, the MYB-bHLH-WD40 (MBW) complex, which is composed of the MYB, basic helix-loop-helix (bHLH), and WD40 repeat families, has been proposed as the main regulatory element for anthocyanin accumulation in Solanaceae. Among the reported regulatory candidates, three MYB1s (SMEL4.1_10g019180.1, SMEL4.1_05g015570.1, SMEL4.1_01g009630.1), biosynthetic activators formerly named *MYB113* [163,164,165,166,167], *SmelMYBL1* (SMEL4.1_10g000420.1, anthocyanin repressor) commonly known as *MYB4*, and a *BHLH42* (SMEL4.1_09g014720.1) [168,169], also called TRANSPARENT TESTA 8 (*TT8*), were included in the defined QGRs (AN1, AN9, AN15, AN17, AN18). Environmental variables, such as light [170,171] and temperature [172], can also affect anthocyanin metabolism. *CRY1* (SMEL4.1_05g017270.1), *COP1* (SMEL4.1_10g002450.1), *SPA3* (SMEL4.1_10g004500.1), the main genes reported to be responsible for light-dependent anthocyanin pigmentation, were associated with QGR AN9 and AN17 [173,174,175,176]. Finally, anthocyanin pigmentation is associated not only with biosynthetic elements, but enzymatic and non-enzymatic factors can interact in the degradation of the pigments, leading to the regulation of anthocyanin discoloration mechanisms [151]. A number of studies have reported potential candidate genes involved in these mechanisms, and the one included in the defined QGRs can be found in Table S3.

## 8. Biotic Resistances (RS)

### 8.1. Pathogens’ Resistance

**Figure 6:**
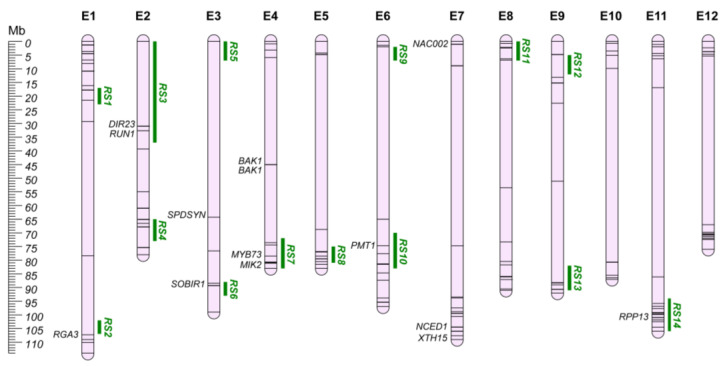
Pathogen resistance QGRs (RS; Green) and pest resistance chromosome map (E1–E12). Main candidate genes are reported in italics, while information about the other candidates (represented by bars on the chromosomes) are shown in Table S3.

World-wide, plant pathogens and pests are among the major effectors in crop production, affecting yield and strongly impacting on social, environmental, and economic costs [177]. As the climate changes, enlarging the geographical area suitable for their establishment and growth, these organisms can spread more easily, requiring new strategies for their control. Eggplant production can be drastically affected by pathogens, with fungal and bacterial wilts representing the main hazards in many parts of the globe. Bacterial wilt is caused by the *Ralstonia solanacearum* species complex, a soil-borne pathogen well adapted to tropical/subtropical regions [178], while fungal wilts are generally caused by *Verticillium dahliae* and *Fusarium oxysporum* f. sp. *melongenae*, mostly causing more than 50% of yield loss [179]. As has occurred in different crops, human selection has caused an erosion of the genetic variability of the cultivated germplasm, leading to a reduction in the number of resistant/tolerant genotypes that have been conventionally applied in breeding programs [180]. For this reason, wild and allied relatives have been employed for the introgression of resistance traits in cultivated eggplants. Data collection revealed a total of 66 QTLs for resistance/tolerance to fungal and bacterial wilt, identifying 14 QGRs (Table S2; Figure 6). Thirty-seven QTLs were not included in the QGRs due to the lack of sufficient information to establish their chromosomal position.

DIR proteins are involved in the biosynthesis of cell wall lignins and lignans, playing a key role in abiotic and biotic stress tolerance [181,182,183,184,185]. A recent genome-wide identification of the eggplant DIR gene family identified potential candidates for biotic resistance, reported to be involved in ROS accumulation and callose deposition in the infection sites (Table S3) [186]. Among them, seven DIRs were included in the defined QGRs, in QTLs associated both with fungal and bacterial wilt.

Four candidate genes for bacterial wilt resistance aligned with the defined QGR regions, both originating from synthenic and transcriptomic approaches. On chr. E4, *MIK2* (QGR RS7; SMEL4.1_04g016080.1) has been reported as maintaining cell wall integrity in resistant genotypes, while *SOBIR1* (QGR RS6; SMEL4.1_03g024090.1) seems to be involved in plant cell death [187]; these two genes activated the pathogen-resistance responses in Arabidopsis [188,189]. Salgon et al. [39] identified tomato synthenic regions for eggplant resistance to *R. solanacearum* (Table S3). By mining them, we identified a putative disease resistance protein RGA from *S. bulbocastanum* (*RGA3*; SMEL4.1_01g035130.1) on chr. E1 (200kb upstream QGR RS2) as potentially responsible for the reported RE-bw resistance locus [190]. Finally, *SmMYB44* (SMEL4.1_04g019540.1; annotated as *MYB73* in v4.1; QGR RS7) has been reported as regulator of the spermidine synthases *SmSPDS* (SMEL4.1_03g012150.1) and *SmSPDS-like* (SME4L.1_06g012830.1, annotated as *PMT1* in v4.1; QGR RS10), leading to spermidine accumulation and resistance to *R. solanacearum* in eggplants [191].

For fungal wilt resistance, two QGRs were identified on chr. E2 (QGR RS3) and E11 (QGR RS14). In QGR RS3, dirigent protein 23 (*DIR23*; SMEL4.1_02g003080.1) was proposed by Barchi et al. [37] as associated with the FomE02.01 resistance locus. This locus was recently investigated by Tassone et al. [192] through the BSAseq approach, assessing the introgression of the resistance locus from *S. aethiopicum*. Thanks to the availability of the eggplant pangenome [68], ten potential candidate genes were identified on the *S. aethiopicum* genome. Among them, *RES1* was described as a putative TMV resistance protein N-like (Solyc02g032200.2) [43]. This gene was annotated as a disease resistance protein *RUN1* (SMEL4.1_02g003050.1) in the 4.1 version of the eggplant genome. RUN1 proteins have been reported to be involved in ROS accumulation and callose deposition in the infection sites after pathogens’ inoculum, providing resistance to fungal penetration in the tissue, and further absence of hypha proliferation [193]. On chr. E11 (QGR RS14), three candidates, including a putative late-blight resistance protein (*R1C-3*; SMEL4.1_00g001090.1) and two homologs of *RPP13*, were selected by Tassone et al. [192] as mainly responsible for the FomCH11 locus. The latter were annotated as proteins of unknown function in the last version of the reference genome, while the annotation of the other six candidates (Table S3) in version v4.1 was consistent with the one reported in the literature.

### 8.2. Pests Resistance

Insects and nematodes can drastically affect plant productivity, causing a wide range of damage both to the vegetative tissues and the berries [194,195]. The genetic basis of eggplant resistance/susceptibility to pests has been poorly investigated, producing only a few transcriptomic works on the topic. Recently, eggplant and tomato were compared for their biochemical and transcriptomic reaction to *Tuta absoluta* (Meyrick) attack [196]. This insect is a leaf miner whose invasion is seriously threatening the commercial tomato industry, easily spreading on other Solanaceae [197]. Multi-omics analysis have been performed in tomato, the main model host for the pest, identifying a signaling cascade mediated by the JA complex as first transcriptional changes upon infection, followed by the activation of genes involved in trichomes’ growth and the biosynthesis of terpene volatiles and phenylpropanoids [198]. In eggplant, the transcriptomic analysis suggested gene regulation in ER protein processing and phenylpropanoid biosynthesis as main responsible in the inhibition of *T. absoluta* infestation (Table S3).

Root-knot nematodes (RKNs, *Meloidogyne* spp.) are endoparasites that attack many cultivated plants, seriously threatening global food safety and production [199]. The southern root-knot nematode *Meloidogyne incognita* is one of the main eggplant nematode parasites [200]. Under invasion conditions, plants react with a wide range of defense mechanisms, including phytohormone biosynthesis (e.g., auxin, cytokinins, salicylic acid, jasmonate, gibberellin, abscisic acid, and brassinosteroids) and modifications in the cell wall composition [201,202]. Zhang et al. [203] investigated the transcriptomic changes in the gene expression of *Solanum torvum* (Sw.), reported to be less susceptible to the nematode, and eggplant under *M. incognita* infestation, and reported several DEGs potentially associated with the pathogen tolerance and susceptibility (Table S3). Among them, 13 nucleotide-binding site–leucine-rich repeat (NBS-LRR) resistance genes were upregulated in eggplant, suggesting their role in the plants’ reaction to pathogen-related damages. Interestingly, *NCED1* (SMEL4.1_07g020880.1), the key enzyme in the defense response mediated by ABA biosynthesis, was upregulated in *S. torvum*, together with two BAK1s (SMEL4.1_04g008770.1 and SMEL4.1_04g008780.1), associated with brassinosteroids biosynthesis, mainly proposed to interact with RKN effectors during invasions [204]. Finally, two xyloglucan endotransglucosylases, associated with structural changes in cell wall expansion, essential for nematode feeding sites’ formation [205], were repressed in *S. torvum* (*XTH15*-SMEL4.1_07g020690.1-and *NAC002*-SMEL4.1_07g000510.1).

## 9. Abiotic Resistances

**Figure 7:**
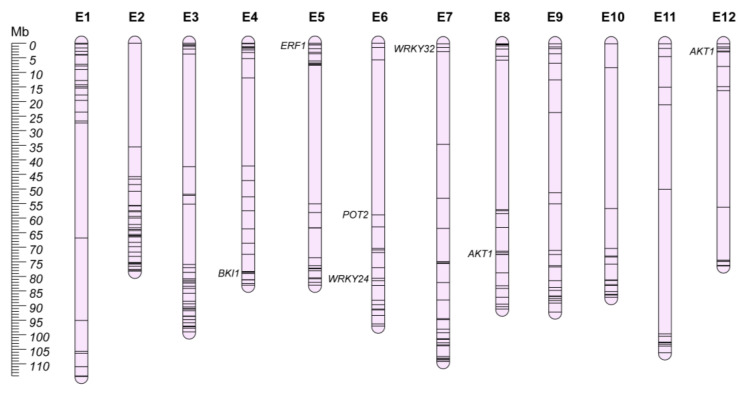
Abiotic resistance chromosome map (E1–E12). Main candidate genes are reported in italics, while information about the other candidates (represented by bars on the chromosomes) is shown in Table S3.

It is clear that the breeding focus in the next few years will be targeted to tolerance and resistance to the main biotic and abiotic stresses, especially in the climate change scenario. Modern eggplant varieties are generally susceptible to several abiotic stresses, including drought, salinity, low and high temperatures, and soil toxicity [206,207,208,209,210]. Thus, a deeper knowledge of the genetic mechanisms involved in the tolerance of such stresses is required to develop new breeding materials able to face and rapidly recover from suboptimal growing conditions. While classical mapping studies and GWA panels have poorly investigated the genetic elements providing tolerance to the main abiotic stresses, great efforts have been focused on transcriptome analysis of sensitive/tolerant accessions under different environmental conditions. Recently, Toppino et al. [211] reviewed in depth the available material for abiotic stresses in eggplant. Here, we provide a selection of the main candidate genes to be explored in the development of novel high-value eggplant cultivars (Table S3; Figure 7).

### 9.1. Osmotic Stress

Osmotic stress represents one of the most important environmental aspects that can negatively impact crop growth and productivity [212], causing an increase in carotenoid and proline content [213]. Drought and water scarcity also negatively affects nitrogen, phosphorus, and potassium uptake, decreasing total soluble solids (TSS), increasing total phenols, superoxide dismutase (SOD), glutathione reductase (GR), electrolyte leakage, pH, and vitamin C [214]. Photosynthetic pigments’ reduction, together with proline, malondialdehyde, total phenolics, and total flavonoids’ accumulation have been reported to be the main effects associated with the water stress in eggplant [210]. Not only water scarcity, but also other environmental conditions (e.g., high salinity and temperatures, land flooding and soil contamination) can produce osmotic stress in plants [215,216,217,218]. For this reason, a wide range of common expression patterns have been observed in reaction to different abiotic stressors [219,220,221]. Stress-associated proteins (SAP), NAC transcription factors, apetala2/ethylene responsive factor (AP2/ERF), and DNA methyltransferases have been reported to be constantly upregulated in eggplant under abiotic stress conditions (Table S3) [222,223,224,225,226,227], while C-repeat binding factors (CBFs) have been proposed as early-stage effectors in the plant response to osmotic and cold stress [228]. For instance, the role of *SmERF1* (SMEL4.1_05g001670.1) was validated under salinity stress by virus-induced gene silencing assay (VIGs), enhancing susceptibility to abiotic stress and downregulating expression levels of other stress defense-related genes [225].

### 9.2. Salt Toxicity Stress

Soil salinity has a negative impact on plant growth, fruit quality, and yield [229] and eggplant has been reported to be moderately susceptible to salinity when compared to other Solanaceae [230]. Different studies have reported an association between salt-induced growth reduction and high accumulation of Na^+^ and Cl^−^ in both roots and shoots, causing stomata closure and increasing leaf turgor potential [231,232]. Furthermore, calcium (Ca^2+^) and potassium (K^+^) concentrations, water consumption, and the K^+^/Na^+^ ratio have been reported to decrease under salinity stress [232,233]. Overall, an excess of NaCl appears to reduce seed germination [234], roots and shoots’ growth, chlorophyll content, and the photosynthetic rates, ending in a reduction in fruit yield [235]. At present, the genetic control of plant reaction to salt accumulation has been poorly investigated, but salts transport mechanisms appear to play a key role in cell detoxification [236], and transcriptome analysis identified a series of transcription factors and structural genes associated with K^+^ and Na^+^ homeostasis (Table S3) [233]. Among them, the interaction of *SmAKT1* (SMEL4.1_08g015230.1 and SMEL4.1_12g001280.1) and *SmSOS1* (annotated as *POT2*; SMEL4.1_06g009410.1) was proposed to regulate Na^+^ transport and accumulation in leaves.

### 9.3. Heat Stress

As the climate is gradually becoming warmer, seedlings’ growth, flower development, fruit set and growth can be drastically compromised by high temperatures that can be scored during summer periods [237]. Furthermore, as high temperatures fasten up fruit ripening leading to significant decreases in total anthocyanin content, heat stress can also harm fruit quality [238,239,240]. Under heat stress, plant cells respond by inducing the expression of genes encoding heat shock proteins (Hsps), involved in preventing heat-related damage and conferring thermotolerance [241]. Generally, these proteins behave as molecular chaperones, preventing protein misfolding and aggregation, maintaining protein homeostasis in cells [242]. Furthermore, specific transcription factors (i.e., heat shock factors-Hsfs) have been reported to control and regulate Hsps’ expression and activation in the cell [243]. Recently, Gong et al. [244] performed a genome-wide identification of Hsps and Hsfs in eggplant, followed by transcriptomic analysis on two inbred lines, contrasting for heat tolerance. The results highlighted that Hsgs and Hsps, belonging to Hsp60, Hsp70, Hsp90, and Hsp100 protein families, were induced by heat stress treatment in the thermotolerant inbred line (Table S3). Hsp70 and Hsp100 families and Hsf class A and B were previously reported by Zhang et al. [239] and Wang et al. [245] to be differentially expressed under heat stress conditions, together with a number of transcription factors (e.g., MYB, ERF/DREB, NAC; Table S3), suggesting potential candidates for elucidating thermotolerance mechanisms in eggplant.

### 9.4. Cold Stress

Contrasting with summer high temperatures, low temperatures in the early stage of cultivation have been recorded in recent years. Cold stress limits plant growth, development, and production, and eggplant appears to be much more sensitive to it compared with other solanaceous crops [246]. Eggplant grows slowly when the temperature is below 17 °C, suffers rapid physiological disorders below 10 °C, and undergoes chilling injury near 7.2 °C [247]. Furthermore, chilling injuries can occur, causing rapid low-pollen viability, plant aging, fruit skin shrinkage, and calyx deterioration and browning [207,248]. Cold sensitivity has been reported to be enhanced by the effect of brassinosteroids (BR), and *BKI1* (SMEL4.1_04g020080.1), under-expressed in sensitive genotypes, was reported to regulate the low temperature-induced BR signal in eggplant [249]. In addition, transcriptomic analysis revealed that a wide number of DEGs were represented by transcription factor families (e.g., AP2/ERF, C2H2, WRKY, bHLH, NAC, and MYB-related; Table S3), and the downregulation of two WRKY transcription factors-*SmWRKY26* (annotated as *WRKY24* in v.4.1; SMEL4.1_06g016680.1) and *SmWRKY32* (SMEL4.1_07g001740.1) through VIGs increased eggplant sensitivity to cold stress, aggravating injuries caused by low temperature [250].

### 9.5. Heavy Metals Stress

The presence of high concentrations of heavy metals (e.g., cadmium, chromium, lead, and nickel) in the soil may have a toxic effect for eggplant [251,252], leading also to the accumulation of such elements in the fruits [253,254]. While soil toxicity effects in eggplant have not been investigated, *S. torvum* has been used as grafting material to improve Cd toxicity and plant resilience [252,255,256,257]. Recently, Cui et al. [258] investigated the methylation impact of *S. torvum* grafting on eggplant genes involved in sulfur metabolism, associated with a lower accumulation of Cd in aerial tissues, highlighting that grafting regulates S metabolism genes (e.g., *STR*, *MGL*, *CGS*, *SULTR21*, *DCYD*, and *SUR*; Table S3), enhancing S absorption and translocation in plants and modulating Cd accumulation.

### 9.6. Low Nitrate Stress

Nitrogen fertilization affects plant vigor, leaf chlorophyll content, fruit settings, dry matter production, and ascorbic acid content [259], as well as flower number, fruit pH and total solid content, fruit weight, and seed number [260]. The development of cultivars with higher N uptake, translocation, and use efficiency, i.e., nitrogen-use-efficiency (NUE) would lower production costs, and will be one of the main challenges to maintain high yields in a sustainable agriculture. However, limited information on genetic variation for this trait is available for eggplant, whose productivity is highly sensitive to N fertilization [261,262]. For example, transcriptomic analysis on four eggplant lines reported upregulation of light-harvesting complexes (LHCs) genes and ferredoxin–NADP reductases (FNRs) in the N-use genotypes, impacting on photosynthetic efficiency [263]. Furthermore, genes involved in responses to inorganic substances, abiotic stimuli, and chemicals were also differentially expressed between contrasting genotypes (Table S3) [264]. The *WRKY33* (annotated as *WRKY24* in v.4.1; SMEL4.1_06g016680.1) transcription factor have been associated with MAP kinases, *YLS9*, and auxin-responsive family genes upregulation, potentially promoting the development of a more efficient root system, as confirmed by overexpressing the orthologue transcription factor in Arabidopsis.

## 10. Concluding Remarks

In the last few decades, the genetic basis of eggplant traits in modern cultivars and their relatives have been investigated by several publications, but methodological differences have made it difficult to efficiently compare their outputs. QGRs here defined represent the regions that most likely contain genetic elements that regulate eggplants’ phenotypes. However, the presence of large QGRs, probably linked to experimental and methodological limits, suggest a need for further dissection of these regions through high-resolution and fine-mapping approaches. This review summarized the state of the art on the understanding of the genetic mechanisms regulating the main agronomical, qualitative and resistance eggplant trait, and the data here organized might find application in future breeding challenges. The information on QTLs here provided can be employed to assist in marker-assisted breeding programs for introducing high-impact regions into superior germplasm. Furthermore, potential candidate genes found within QTL regions can be selected and their effects can be examined in vivo through techniques such as CRISPR-CAS9 gene editing or transient manipulation of gene expression. This can lead not only to the identification of the genes that control a particular trait, but also to the detection of genetic elements responsible for trait variation. These variations, known as functional markers, are the most efficient molecular markers for marker-assisted selection (MAS) because they are directly linked to the trait and, unlike genetically linked markers, do not require validation in other populations. Indeed, both genetic elements and their interactions can pose challenges in varietal development. Pleiotropic, dominant, and epistatic effects have been documented in the literature for multiple traits. For example, anthocyanin regulation is governed by a complex network of interactions and pleiotropic effects. Guan et al. [265] reported a major QTL responsible for both leaf vein pigmentation and pericarp color that explains over 50% of phenotypic variability, while Salgon et al. [40] identified epistasis affecting polygenic resistance to *R. pseudosolanacearum*, where the epistatic effect accounted for 35.7% of total phenotypic variance. Such interactions, especially those of low impact, have been reported to be potentially biased by background QTLs [266]. Hence, a thorough understanding of the parental genetic background is crucial for the development of trait-focused breeding programs, and relevant information can be obtained from the original articles cited in these reviews.

## Data Availability

Data are available at their original publicly accessible repository.

## Supplementary Materials

The following supporting information can be downloaded at: https://www.mdpi.com/article/10.3390/plants12051016/s1, Table S1: List of the manuscripts reporting QTLs, including information on the experimental populations; Table S2: List of the QTLs reported in literature; Table S3: List of the candidate genes reported in literature.
